# Supplementary material for: Rossby wave second harmonic generation observed in the middle atmosphere
Source: Nat Commun. 2022 Dec 7;13:7544. doi: 10.1038/s41467-022-35142-3 (PMC9729661; doi:10.1038/s41467-022-35142-3)
Supplement: Supplementary file 1 — Supplementary Information [file 41467_2022_35142_MOESM1_ESM.pdf]

Supplementary Information for: Rossby wave second harmonic generation observed in the middle atmosphere

Maosheng He[1*] and Jeffrey M. Forbes[2]

[1*]Key Laboratory of Solar Activity and Space Weather, National Space Science Center, Chinese Academy of Sciences, Beijing, People's Republic of China.

[2] Ann & H.J. Smead Department of Aerospace Engineering Sciences, University of Colorado, Boulder, USA, Boulder, USA.

*Corresponding author(s). E-mail(s): hmq512@gmail.com;
Contributing authors: forbes@colorado.edu;

Figures

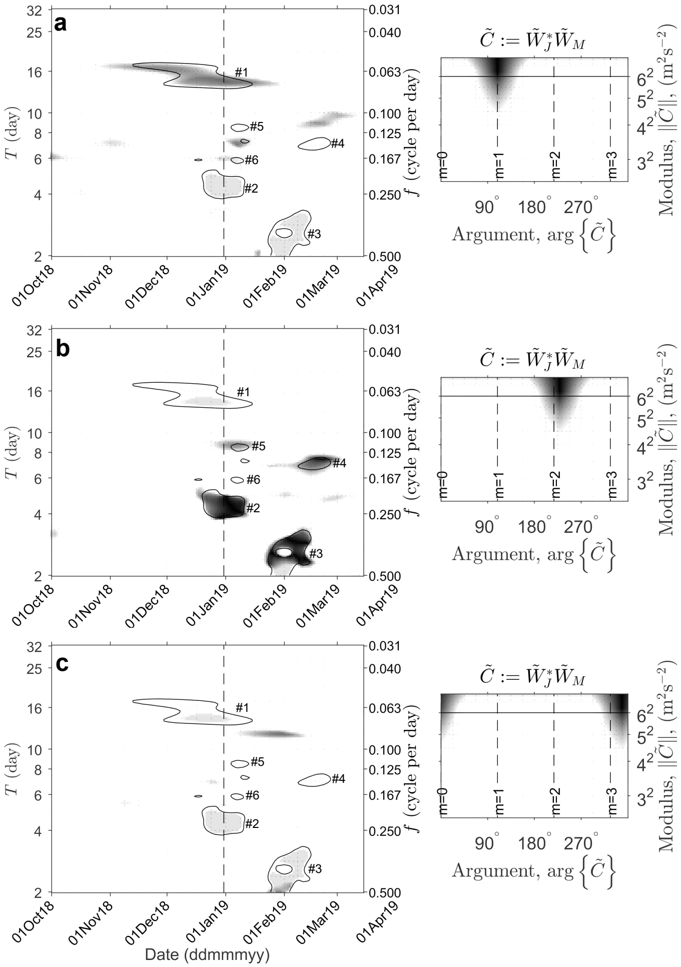

Fig. 1: Color-filtered versions of Fig. 2. **a** Cyan-, **b** magenta-, and **c** yellow-passed.

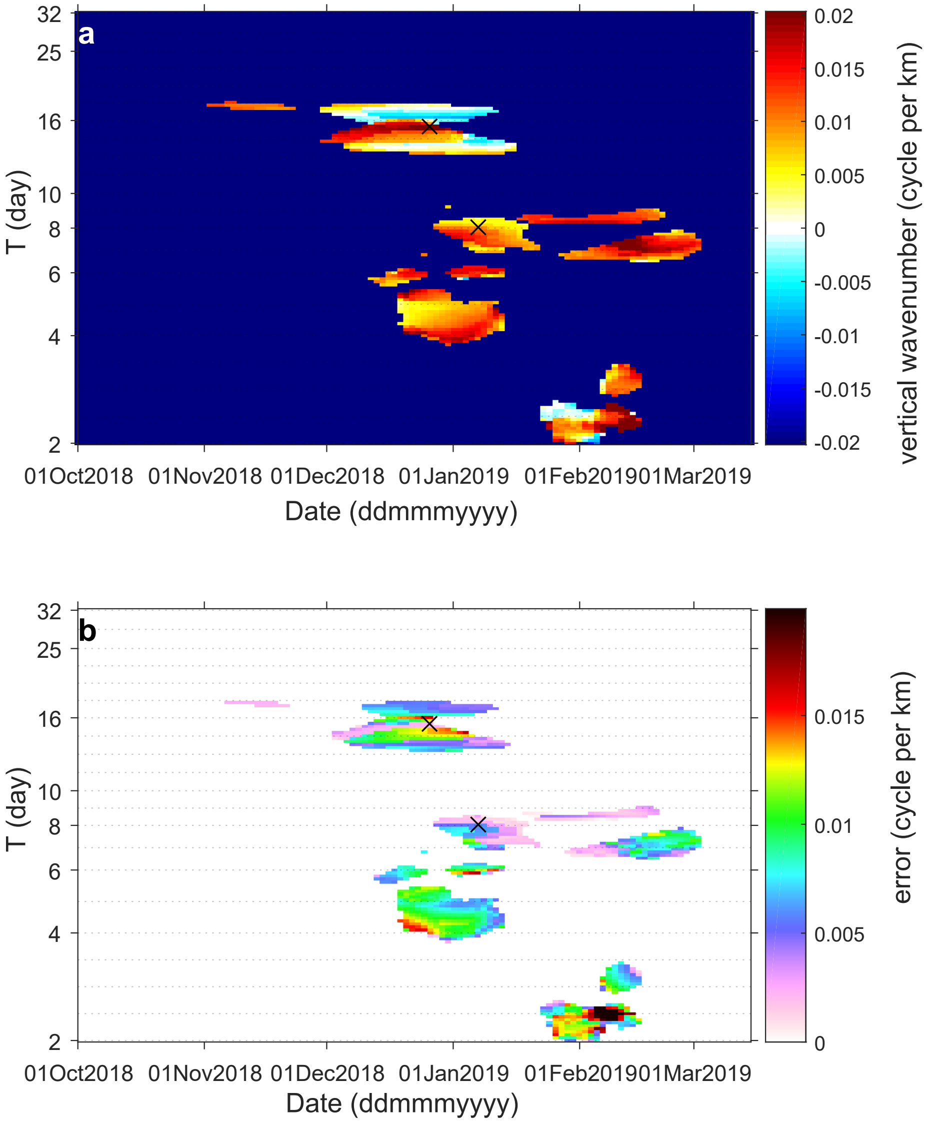

Fig. 2: Vertical wavenumber estimation of waves involved in Fig. 2. **a** The wavenumber. **b** The uncertainty. The crosses indicate two spectral peaks marked in Fig. 1. Saturated out here in dark blue are regions associated with a magnitude below 25 m^2s^{-2} in Fig. 1. See Supplementary Methods for details.

Methods

In Methods, we use the complex amplitudes $\tilde{W}_{J,u,h}$, $\tilde{W}_{J,v,h}$,$\tilde{W}_{M,u,h}$, and $\tilde{W}_{M,v,h}$ to calculate the zonal wavenumber. Here, we use them to calculate the vertical wavenumber. We define $\tilde{D}_h := \tilde{W}_{h+1\mathrm{km}}\tilde{W}_h^*$ where $\tilde{W}_h$ denotes any of the above four variables. Under the assumption that the vertical wavelength of the underlying wave is longer than 2 km, $\frac{\arg\{\tilde{D}_h\}}{2\pi\cdot 1\mathrm{km}}$ is a measure of the vertical wavenumber in the unit cycle per km. For a robust estimation, we replace $\tilde{D}_h$ with its average $\langle\tilde{D}\rangle$ across two longitude sectors (M and J), two components (u and v), and all altitude levels (h =81,...,95 km). Displayed in Supplementary Fig. 2a is the vertical wavenumber $n := \frac{\arg\{\langle\tilde{D}\rangle\}}{2\pi\cdot 1\mathrm{km}}$, for which only $\tilde{D}_h$ above the 0.01 significance level is used.

According to the definition, $\tilde{D}_h\frac{\langle\tilde{D}\rangle^*}{\|\langle\tilde{D}\rangle\|}$ measures the deviation of $\tilde{D}_h$ to its average, its image part $\Im(\tilde{D}_h\frac{\langle\tilde{D}\rangle^*}{\|\langle\tilde{D}\rangle\|})$ measures the tangential deviation, and its standard deviation $\sigma\left\{\Im(\tilde{D}_h\frac{\langle\tilde{D}\rangle^*}{\|\langle\tilde{D}\rangle\|})\right\}$ is used to define an angle $\delta := \arctan\frac{\sigma\{\Im(\tilde{D}_h\langle\tilde{D}\rangle^*)\}}{\|\langle\tilde{D}\rangle\|^2}$ so that $\frac{\delta}{2\pi\cdot 1\mathrm{km}}$ measures the uncertainty of the vertical wavenumber as displayed in Supplementary Fig. 2b.
